# An immune-related signature for optimizing prognosis prediction and treatment decision of hepatocellular carcinoma

**DOI:** 10.1186/s40001-023-01091-w

**Published:** 2023-03-15

**Authors:** Ninghua Yao, Wei Jiang, Yilang Wang, Qianqian Song, Xiaolei Cao, Wenjie Zheng, Jie Zhang

**Affiliations:** 1 Department of Oncology, Affiliated Hospital of Nantong University, Nantong, 226001 People’s Republic of China; 2 Research Center of Clinical Medicine, Affiliated Hospital of Nantong University, Nantong, 226001 People’s Republic of China; 3 Department of Neurology, Affiliated Wuxi No.2 People’s Hospital of Nanjing Medical University, Wuxi, People’s Republic of China; 4 Department of Oncology, Affiliated Tumor Hospital of Nantong University, Nantong, People’s Republic of China; 5 Department of Radiology, Wake Forest School of Medicine, Winston-Salem, USA; 6 School of Medicine, Nantong University, Nantong, 226001 Jiangsu China

**Keywords:** Hepatocellular carcinoma, Immune genes, Signature, Prognosis, Precise therapy

## Abstract

**Background:**

An immune-related gene signature (IGS) was established for discriminating prognosis, predicting benefit of immunotherapy, and exploring therapeutic options in hepatocellular carcinoma (HCC).

**Methods:**

Based on Immune-related hub genes and The Cancer Genome Atlas (TCGA) LIHC dataset (*n* = 363), an immune-related gene signature (IGS) was established by least absolute shrinkage and selection operator (LASSO) analysis. The prognostic significance and clinical implications of IGS were verified in International Cancer Genome Consortium (ICGC) and Chinese HCC (CHCC) cohorts. The molecular and immune characteristics and the benefit of immune checkpoint inhibitor (ICI) therapy in IGS-defined subgroups were analyzed. In addition, by leveraging the Cancer Therapeutics Response Portal (CTRP) and PRISM Repurposing datasets, we determined the potential therapeutic agents for high IGS-risk patients.

**Results:**

The IGS was constructed based on 8 immune-related hub genes with individual coefficients. The IGS risk model could robustly predict the survival of HCC patients in TCGA, ICGC, and CHCC cohorts. Compared with 4 previous established immune genes-based signatures, IGS exhibited superior performance in survival prediction. Additionally, for immunological characteristics and enriched pathways, a low-IGS score was correlated with IL-6/JAK/STAT3 signaling, inflammatory response and interferon α/γ response pathways, low TP53 mutation rate, high infiltration level, and more benefit from ICI therapy. In contrast, high IGS score manifested an immunosuppressive microenvironment and activated aggressive pathways. Finally, by in silico screening potential compounds, vindesine, ispinesib and dasatinib were identified as potential therapeutic agents for high-IGS risk patients.

**Conclusions:**

This study developed a robust IGS model for survival prediction of HCC patients, providing new insights into integrating tailored risk stratification with precise immunotherapy and screening potentially targeted agents.

**Supplementary Information:**

The online version contains supplementary material available at 10.1186/s40001-023-01091-w.

## Background

Liver cancer is one of the most common cancers and a leading cause of tumor-associated mortality worldwide [1]. Hepatocellular carcinoma (HCC) comprises approximately 70% of primary liver cancers, which may be caused by hepatitis B virus (HBV) or hepatitis C virus (HCV) infection, alcohol abuse, non-alcoholic steatohepatitis (NASH) and metabolic disorders. Typical first-line treatment for early-stage patients is surgical resection [2, 3]. However, up to 70% of patients are diagnosed at internal or advanced stages, for whom liver transplantation, transcatheter arterial chemoembolization (TACE), radiotherapy, chemotherapy, and systematic therapy are optional strategies [4]. In recent years, some targeted agents (e.g., sorafenib, lenvatinib, and regorafenib), have shown benefits for treating metastatic or unresectable HCC. Despite the benefit from the progress in therapies, the overall survival of advanced HCC patients is still unsatisfactory [5]. In this scenario, immunotherapy has emerged as a promising treatment for inhibiting tumor progression and even relapse [6].

Immune checkpoints have been implicated in tumor progression and correlated with clinical outcomes of tumor patients with immunotherapy. Currently, increasing studies suggested that immune-checkpoint blockade (ICB) might be a promising option for inoperable HCC cases [7]. ICB therapeutics targeting programmed cell death 1/ligand 1 (PD-1/PD-L1) and cytotoxic T lymphocyte associated protein 4 (CTLA-4) have shown efficacy in a variety of T cell–inflamed cancers [8]. In addition, the combination of PD-1/PD-L1 and CTLA-4 inhibitors significantly improved objective response rates (ORRs) and patients’ survival [9]. However, the ORRs of ICB treatment are relatively low in less-T cells- inflamed HCC [10]. Based on the integrative genomic and transcriptomic investigations, the immunosuppressive tumor microenvironment (TME) might contribute to the weak response of ICB in HCC patients [11].

As a number of studies have suggested the crucial roles of the immune-related genes in cancer progression and immune-therapeutic response, it is of great significance to identify novel immune biomarkers and potential targets that throw light on improving the prognosis of HCC patients [12]. Currently, the publicly available large-scale cancer omics datasets provide us multiple strategies to identify candidate tumor biomarkers and potential targets. In this study, we developed and validated a robust risk model for HCC, which could predict the prognosis of HCC patients and the response of immunotherapy. The immune-related hub genes with prognostic significance were identified to construct an immune-related gene signature (IGS) based on The Cancer Genome Atlas (TCGA) training cohort, which was further validated in the internal TCGA testing cohort and external HCC cohorts. We then characterized the immune features of HCC patients at high- or low- IGS risk and evaluated the prognostic capacity of IGS in patients treated with immunotherapy. In addition, potential therapeutic agents were screened to potentially enhance the response of immunotherapy and to improve the survival of HCC patients with high IGS risk. The current study suggested that IGS was a promising biomarker for predicting survival and immunotherapy response of HCC patients.

## Materials and methods

### Datasets information and data processing

To identify and verify the prognostic significance of IGS risk model, we included three HCC publicly available cohorts (Additional file 1: Table S1). RNA-seq profiles for 363 HCC samples and 50 normal liver samples were obtained from TCGA-LIHC dataset (https://portal.gdc.cancer.gov/repository), which were subsequently converted into the Transcripts Per Million (TPM) values. Survival data were achieved from TCGA Pan-Cancer Clinical Data Resource (TCGA-CDR). TPM normalized expression profiles of 226 normal liver tissues were downloaded from GTEx database. Liver cancer-RIKEN, JP project (LIRI-JP) included Gene profiles, somatic mutation data and clinical information of 229 HCC samples were downloaded from the International Cancer Genome Consortium (ICGC) portal (https://dcc.icgc.org/projects/LIRI-JP). Normalized gene profiles and survival data of Chinese HCC (CHCC) cohort with 159 HCC tissues and adjacent tissues patients of Zhongshan Hospital (Shanghai, China) were downloaded from NODE (https://www.biosino.org/node) [13]. Somatic mutations were analyzed using R package ‘maftools’. Baseline patient characteristics of the three cohorts above were summarized in Additional file 1: Tables S2, S3 and S4. Two immunotherapeutic cohorts were included in this study to test the efficacy of IGS score in predicting immunotherapeutic response. The gene expression and clinical information of patients with locally advanced or metastatic urothelial carcinoma administrated with anti-PD-L1 antibody atezolizumab were obtained from IMvigor210 cohort (http://research-pub.Gene.com/imvigor210corebiologies) [14]. The raw gene expression data of IMvigor210 were normalized and transformed into TPM values using R packages “arrayQualityMetrics”, “voom”, and “limma”, respectively. The gene profiles and detailed clinical information of metastatic melanoma patients treated with an anti-PD-1 antibody pembrolizumab were achieved from GSE78220 cohort. The background adjustment, normalization, and logarithmic processing were performed using R package “affy” [15]. The list of immune-related genes was achieved from ImmPort database (https://immport.org/shared/home).

### Establishment and validation of the immune signature

The differentially expressed genes (DEGs) between 363 HCC tissues (TCGA-LIHC database) and normal liver tissues (50 samples from TCGA-LIHC database and 226 samples from GTEx database) were identified using the R package “DESeq2” with a threshold of *P* < 0.05 and Fold change > 2. The entire TCGA HCC cases (*n* = 363) were randomly divided into a training cohort (*n* = 255) and testing cohort (*n* = 108) in a ratio of 3:1. Following overlapping the DEGs with immune-related genes from ImmPort database, the immune-related hub genes were further screened using univariate Cox regression analysis with a threshold of *P* < 0.05. Subsequently, the LASSO algorithm and 200-fold cross-validation were performed to construct the immune genes-based signature (IGS) model using R package “LASSO” and “cv.glmnet” in the training cohort. The IGS score of each sample was calculated as IGS score = ∑_i=1,2,…n_ (coefficients(gene_i_) × gene_i_ expression). Based on the median value of IGS score, the patients in each cohort were stratified into high-risk and low-risk groups. The efficacy and predictive capacity of IGS was evaluated by the time-dependent receiver operating characteristic (ROC) curve and Kaplan–Meier (KM) curve by performing R package “survivalROC” and R package “survival”, respectively. Univariate and multivariate Cox regression analyses were performed to evaluated the IGS score as an independent prognostic factor for HCC OS. In additional to TCGA internal validation cohort and entire validation cohort, the IGS model was also validated in ICGC cohort and external CHCC cohort. A prognostic nomogram model was then established with calibration curve based on the IGS score and clinical parameters by performing “rms” and “nomogramEx” R packages. Four previously published immune-related signatures were referred to compare the predictive efficacy and accuracy in CHCC cohort [16–19].

### Immune cell infiltration and enrichment analyses

The infiltration of immune cells was quantified by performing CIBERSORT algorithm [20]. The infiltration scores of different groups were calculated by ImmuCellAI algorithm [21]. The correlation of IGS score with immune-related pathways was evaluated using single sample gene set enrichment analysis (ssGSEA). Gene Ontology (GO) and Kyoto Encyclopedia of Genes and Genomes (KEGG) were performed to conduct enrichment analysis. GSEA was performed to detect the difference in DEGs between the high-risk and low-risk group in the enrichment of the MSigDB Collection (h.all.v7.2.symbols.gmt). Adjusted *P*-value less than 0.05 was considered statistically significant. Somatic variant analysis was used to investigate differentially mutated genes associated with the ICI response by performing “maftools” R package [22].

### Drug sensitivity analyses

Gene profiles of cancer cell lines were downloaded from the Broad Institute-Cancer Cell Line Encyclopedia (CCLE) project (https://portals.broadinstitute.org/ccle/). Drug sensitivity data of CCLs were obtained from the Cancer Therapeutics Response Portal (CTRP v.2.0, https://portals.broadinstitute.org/ctrp, 481 compounds) and PRISM Repurposing dataset (19Q4, https://depmap.org/portal/prism, 1448 compounds). Missing area under the dose–response curve were imputed using Knearest neighbor (k-NN). Before imputation, compounds with more than 20% of missing data were excluded. Molecular profiles achieved from CCLE dataset were used for further CTRP and PRISM analyses.

### Statistical analysis

The statistical tests analyses in this study were performed in R v3.6.1 software (Vienna, Austria). Student’s *t*-test, one-way analysis of variance, Wilcoxon rank-sum test or Kruskal–Wallis test were performed to compare the differences of continuous variables. The chi-square test was used for comparison of categorical variables. Correlation analysis was computed by performing Pearson algorithm. *P*-value < 0.05 was considered of statistical significance.

## Results

### Construction and validation of an immune-related prognostic signature for overall survival in HCC patients

By comparing 363 HCC cases with 50 normal liver tissues in TCGA data portal and 226 normal tissues in GTEx database, 2371 significantly up-regulated genes and 544 down-regulated genes were identified with a threshold of *P* < 0.05 and Fold change > 2 (Additional file 1: Fig S1A, B). Following intersection to 1811 immune-related genes derived from the ImmPort database, 251 immune-related hub genes were extracted from the DEGs (Additional file 1: Fig S1C). Enrichment analysis indicated that these genes were implicated in immune and TME-related pathways including cell chemotaxis, antigen processing and presentation, receptor ligand activity, signaling receptor activator activity, cytokine activity, MHC protein complex, cytokine-cytokine receptor interaction, and chemokine signaling pathway (Additional file 1: Fig S1D, E).

**Fig. 1:**
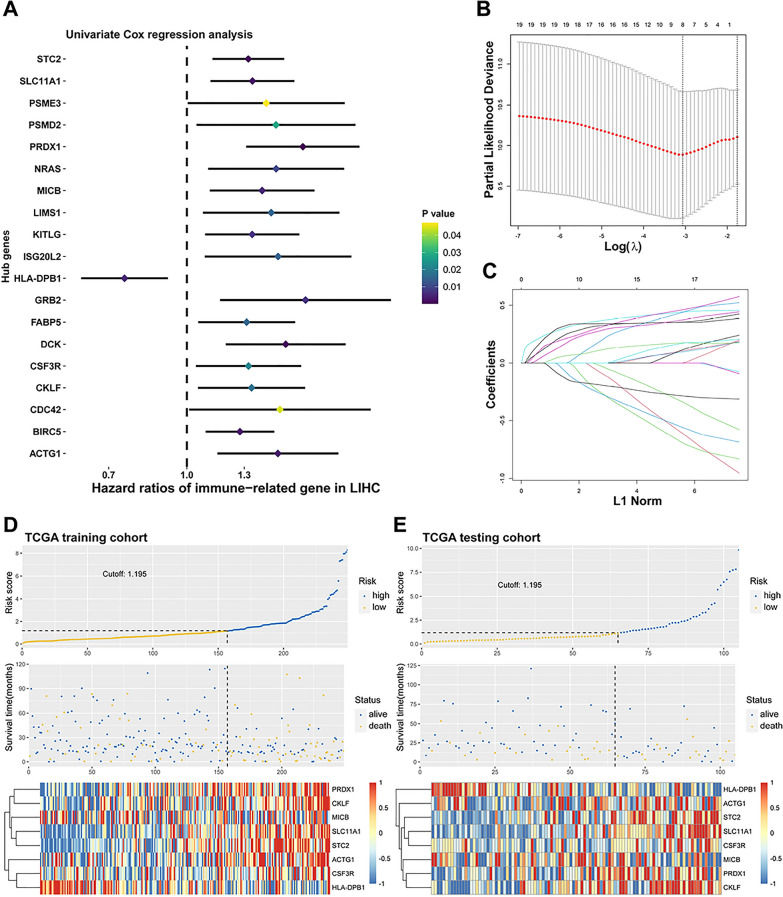
Establishment of the immune-related gene signature for HCC patients. **A**, the Forest plot demonstrating the results of Univariable Cox analysis with statistical significance of the immune related-DEGs in TCGA training cohort. **B**, **C**, the LASSO Cox regression algorithm was performed to identify the most robust prognostic genes in the 19 immune related-DEGs. **D**, The distribution of the risk score, survival status, and the 8 IGS genes for patients in low- and high-risk groups in TCGA training cohort. **E**, the distribution of the risk score, survival status, and expression of the 8 IGS genes for patients in TCGA testing cohort

Subsequently, HCC cases of TCGA dataset (363) were randomly divided into a training cohort (255) and testing cohort (108) in a 7:3 ratio. Of these immune-related hub genes, 19 candidate genes were further defined as significantly relevant with OS of HCC patients by performing Univariate Cox regression analysis (threshold: *P* < 0.05, HR > 1.25 or < 0.75; Fig. 1A) in the TCGA training cohort. The LASSO Cox algorithm was then conducted to identify the most robust prognostic genes among the immune-related DEGs in the TCGA training cohort. Following 200-fold cross-validation for variable selection, an optimal *λ* value of 8 was selected (Fig. 1B, C). Then, 8 Immune-related genes with individual coefficients were assembled to establish an Immune-related gene signature (Additional file 1: Fig S2A, B). Based on the IGS, an immune-related prognostic risk score normalized to Z-score was calculated for each patient in the TCGA training cohort and testing cohort (Fig. 1D, E).

### The IGS presented excellent performance in predicting the overall survival of HCC patients

**Fig. 2:**
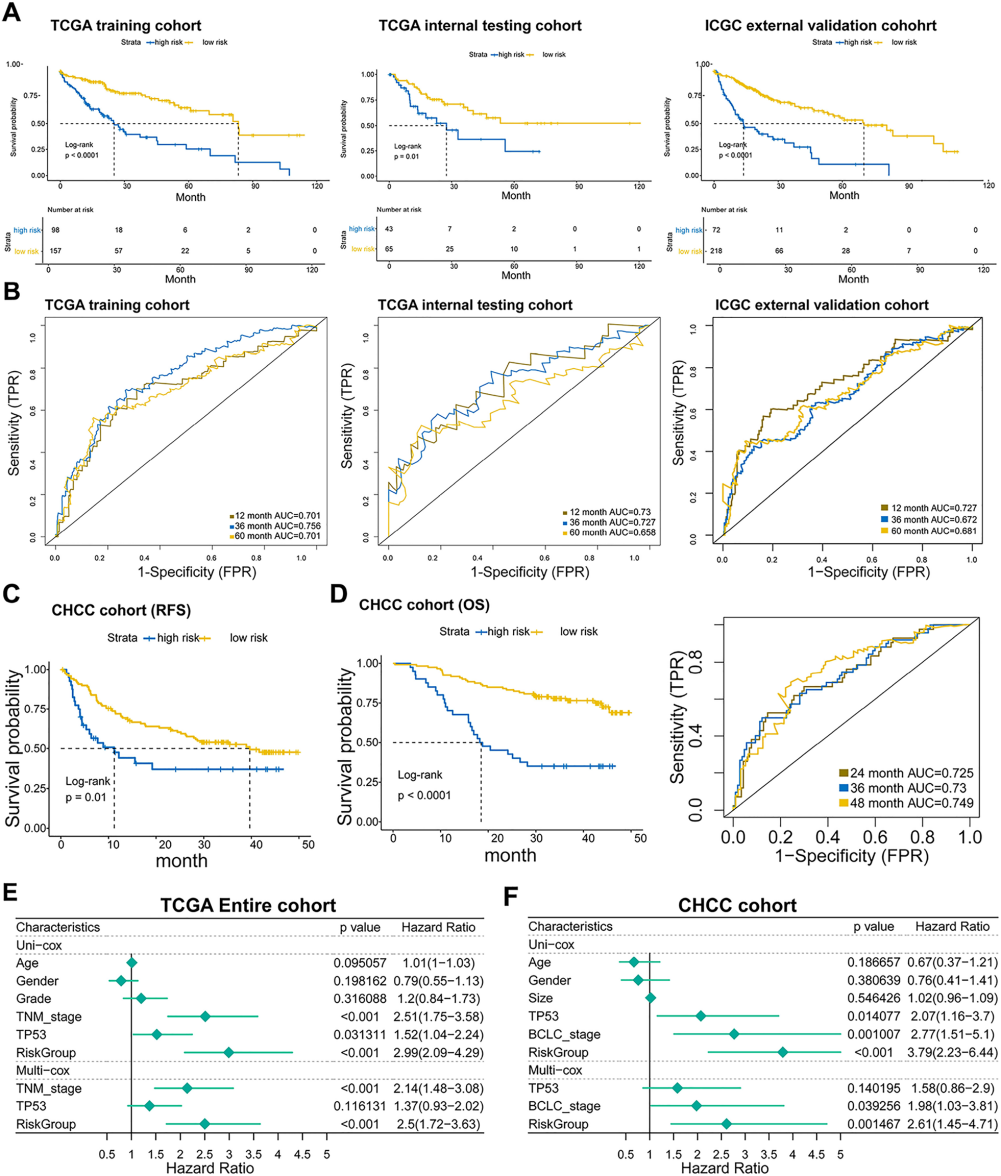
IGS risk model accurately predicted survival of HCC patients. **A**, the prognostic value of IGS was validated in the TCGA training cohort, TCGA internal testing cohort, and external ICGC cohort by Kaplan–Meier analysis. **B**, time-dependent ROC curves of IGS in the three cohorts above. **C**, the DFS of the high or low IGS risk group in the CHCC cohort. **D**, the OS curve and time-dependent ROC curves with high or low IGS risk group in CHCC cohort. **E**, The Univariate and Multivariate Cox regression analyses were performed on 6 variables, including IGS risk score, Age, Gender, Grade, TNM stage, and TP53 status in the pooled TCGA cohort. **F**, the Univariate and Multivariate Cox regression analyses with IGS risk score, Age, Gender, tumor size, BCLC stage and TP53 status were performed in the CHCC cohort

Then, we evaluated the prognostic performance in different HCC cohorts. Based on the median value of the IGS score, each HCC cohort was stratified into a high-risk group and a low-risk group. As illustrated in Fig. 2A, HCC patients in high-IGS-risk group exhibited worse OS than that of low-IGS-risk group, which was coherently determined in TCGA training cohort (HR = 3.35, 95% CI = 2.19–5.13, *P* < 0.0001), TCGA internal testing cohort (HR = 2.25, 95% CI = 1.19–4.23, *P* = 0.01), and ICGC cohort (HR = 2.97, 95% CI = 2.09–4.22, *P* < 0.0001). Consistently, the time-dependent ROC curves indicated that the IGS risk model also displayed excellent AUC in the three cohorts at the time point of 1 year (training cohort, 0.701; testing cohort, 0.73; ICGC cohort, 0.727), 3 years (training cohort, 0.756; testing cohort, 0.727; ICGC cohort, 0.672), and 5 years (training cohort, 0.701; testing cohort, 0.658; ICGC cohort, 0.681), suggesting a favorable predictive capacity of the IGS risk model in short- and long-term follow-up of HCC patients (Fig. 2B).

In addition, the CHCC cohort, as an external independent HCC cohort, was used to further validate the prognostic value of the IGS risk model (Fig. 2C, D). HCC patients with high IGS risk score had poorer RFS (HR = 1.88, 95% CI = 1.15–3.06, *P* = 0.01) and OS (HR = 3.79, 95% CI = 2.23–6.44, *P* < 0.0001) than patients with low IGS risk score. It is worth noting that the time-dependent ROC curve also indicated that the IGS risk model had satisfactory performance in predicting OS in CHCC cohort patients at different time periods (2 year, 0.725; 3 year, 0.73; 4 year, 0.749). Subsequently, Univariate and Multivariate Cox regression analyses were conducted in the pooled TCGA cohort including IGS risk score and 5 clinical variables (Age, Gender, Grade, TNM stage, and TP53 status). The results revealed that TNM stage (*P* < 0.001) and IGS risk score (*P* < 0.001) were independent risk factors for OS of HCC patients (Fig. 2E). For the CHCC cohort, Age, Gender, tumor size, TP53 status, IGS risk score, and BCLC stage were enrolled into the COX regression analyses. It arrived at a similar conclusion that the TNM stage (*P* = 0.039) and IGS risk score (*P* = 0.015) were independent risk factors for HCC prognosis (Fig. 2F).

Subsequently, we conducted stratification analyses to evaluate the prognostic significance of the IGS risk model for HCC patients. The pooled TCGA or CHCC cohort was stratified into sub-groups based on clinical features such as Age (> = 60 or < 60), Gender, Stage (I/II or III/IV), and TP53 status (wide-type or mutated). In both of the two independent cohorts, the IGS risk model could profoundly predict the OS of HCC patients in all sub-groups (Additional file 1: Fig S3A, B). It suggested that IGS retained its prognostic value in differentiating high risk cases in different subgroups.

### IGS-based nomogram and comparison with other immune-related signatures

**Fig. 3:**
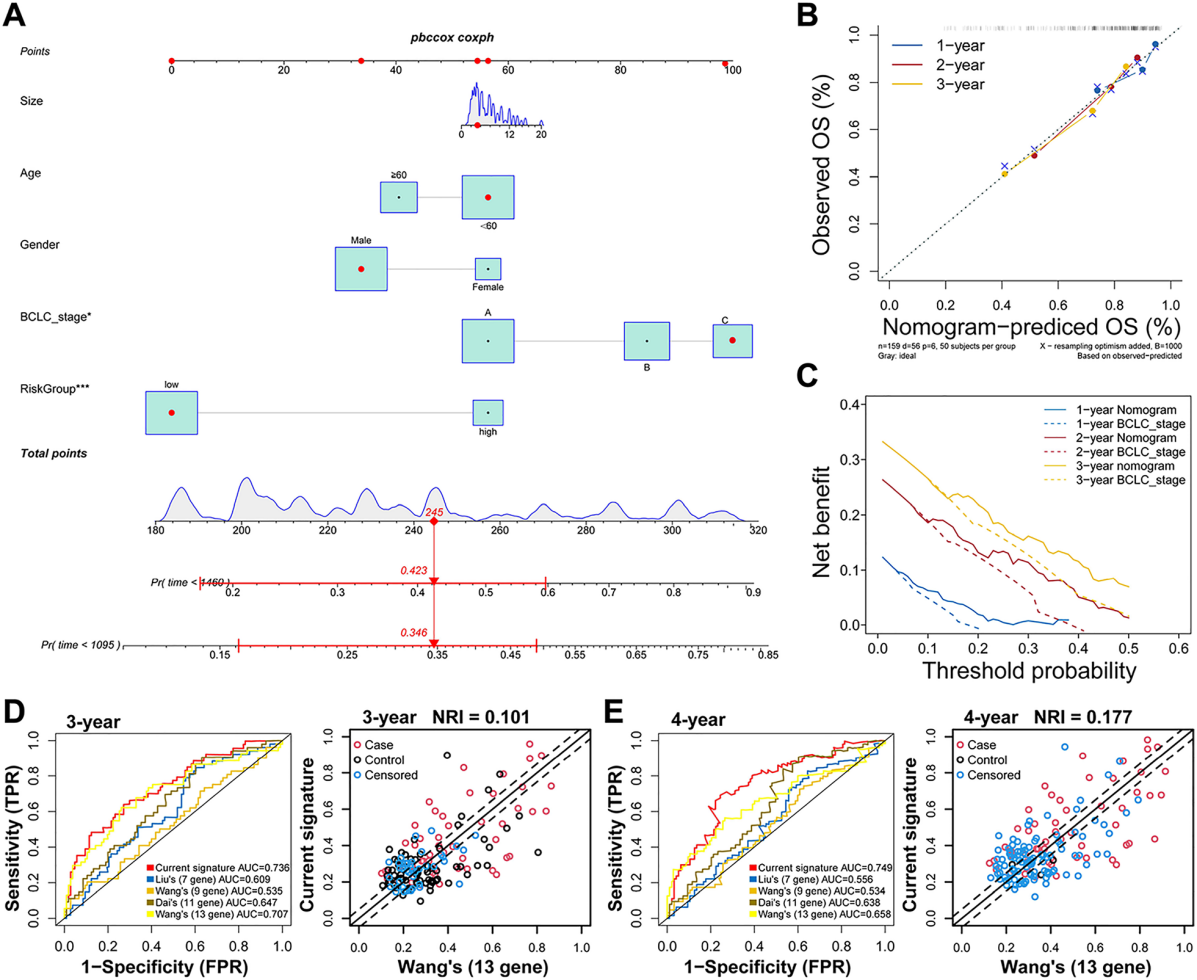
Construction of nomogram and the comparison with previous immune-related signatures in CHCC cohort. **A**, Nomograms for predicting patients with OS after surgery in CHCC cohort. **B**, Calibration plot of agreement between predicted by nomogram and observed 2 year, 3 year, and 4 year outcomes. **C**, Time-dependent DCA curves. **D**, ROC of IGS and 4 previous immune-related signatures in CHCC cohort in 3-year follow-up. The comparison of NRI (Net Reclassification Index) between IGS and Wang’s 13-gene signature in 3-year follow-up. **E**, ROC of IGS and 4 previous signatures in CHCC cohort in 4-year follow-up. The comparison of NRI between IGS and Wang’s 13-gene signature in 4-year follow-up

Based on the prognostic performance of the IGS risk model, we established a nomogram including IGS risk score and clinicopathological features such as age, tumor size, BCLC stage and gender in the CHCC cohort (Fig. 3A). As demonstrated in the calibration plot, the prediction lines of the nomogram for 1-, 2-, and 3-year survival probability were extremely close to the 45-degree reference line, suggesting the profoundly predictive accuracy for HCC patients (Fig. 3B). In addition, in contrast to the BCLC stage, the nomogram exhibited superior performance in predicting the OS of HCC patients (Fig. 3C). Furthermore, we compared the performance of the IGS risk model with 4 previous immune-related signatures in the CHCC cohort. At the time point of 3 year, the AUC of IGS (0.736) was obviously higher than that of Liu’s 7-gene signature (0.609), Wang’s 9-gene signature (0.535), Dai’s 11-gene signature (0.647), and Wang’s 13-gene signature (0.707), respectively. Moreover, the net reclassification improvement (NRI) was conducted to quantify the improvement in predictive performance, which showed a 10.1% improvement to Wang’s 13-gene signature by performing the IGS risk model (Fig. 3D). Consistently, with an NRI of 17.7%, the IGS risk model also had better performance in 4 year’s follow-up (0.749 vs. 0.556, 0.534, 0.638, and 0.658) compared with Wang’s 13-gene signature (Fig. 3E).

### The genomic alterations and mutational features in different IGS groups

**Fig. 4:**
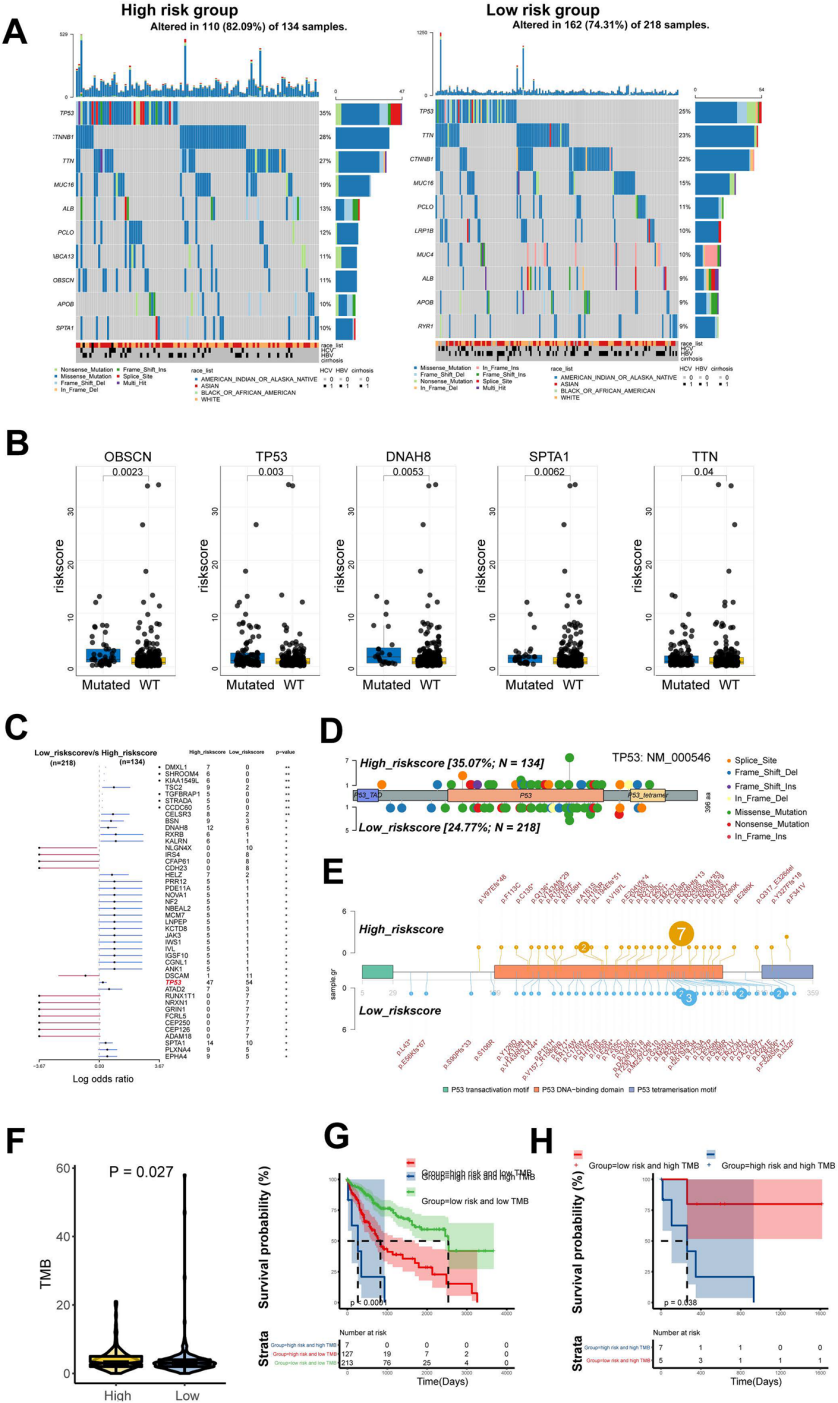
The mutational features of the IGS-high and IGS-low groups. **A**, the mutational landscapes of the high IGS risk subgroup and low IGS risk subgroup. **B**, the risk score of HCC patients with wide-type and mutation status of 5 genes with most common mutations. **C**, representative mutated genes in the samples of IGS-high and IGS-low subgroups. **D**, **E**, a lollipop plot illustrated the different mutation position of TP53 between the IGS-high and-low subgroup. **F**, the TMB in the IGS-high or -low sub-group in the pooled TCGA cohort. **G**, The OS of HCC patients with high IGS risk/high TMB, low IGS risk/low TMB, and high IGS risk/low TMB. **H**, Kaplan–Meier analysis of high IGS risk and low IGS risk in HCC patients with high TMB

With a threshold of |log2(Fold Change)|≥ 1 and *P* < 0.01, 187 DEGs were identified in high- IGS risk samples and low- IGS risk samples of the TCGA cohort, respectively (Additional file 1: Fig S4A). Subsequently, these DEGs were subjected to GO enrichment analysis to predict their potential roles in biological processes. The result showed that they were implicated in pathways including response to stimulus, immune system process, metabolic process, cellular process, and multi-organismal process (Additional file 1: Fig S4B). In addition, GSEA was also conducted to investigate the IGS genes-related pathways in TCGA pooled cohort. The gene sets of the IGS-high samples were mainly enriched in DNA repair, E2F target, G2M checkpoint, and MYC targets (Additional file 1: Fig S4C), while the IGS-low group was correlated with IL-6/JAK/STAT3 signaling, inflammatory response and interferon α/γ response (Additional file 1: Fig S4D). Next, we identified the top 10 most frequently mutated genes in the IGS risk score-stratified subgroups. In both subgroups, the mutation rates of TP53, TTN, CTNNB1 and MUC16 were higher than 15%. The mutations of the ABCA13, OBSCN and SPTA1 genes were more frequent in the IGS-high subgroup. In contrast, the mutations of LRP1B, MUC4 and RYR1 were more commonly observed in the IGS-low subgroup (Fig. 4A). Accordingly, the IGS score of patients with OBSCN, TP53, DNAH8, SPTA1, or TTN mutation was significantly higher than that of wild-type patients (Fig. 4B). Interestingly, as shown in Fig. 4C, TP53 ranked the highest mutation frequency in both of the IGS-high and -low sub-group. More specifically, distinct mutation spots of TP53 were detected in the two sub-groups according to the lollipop analysis (Fig. 4D, E). Furthermore, the tumor mutational burden (TMB) was significantly elevated in patients with higher IGS risk score (*P* = 0.027; Fig. 4F). Moreover, the IGS score was negatively correlated with PDL1 expression in TCGA data portal (Additional file 1: Fig S5A). Interestingly, the protein expressions of MLH1 and MSH6, two typical MSI genes, were significantly elevated in high IGS risk group in CHCC cohort, suggesting the potentially promoting roles in TMB (Additional file 1: Fig S5B). As illustrated in Fig. 4G, patients with high IGS risk score/high TMB had shorter OS than patients with low IGS risk score/low TMB and high IGS risk score/low TMB (*P* < 0.001), respectively. In addition, for patients with high TMB, higher IGS score also suggested a poorer prognosis (*P* = 0.038; Fig. 4H).

### Immune characteristics of HCC patients with high- or low- IGS score

**Fig. 5:**
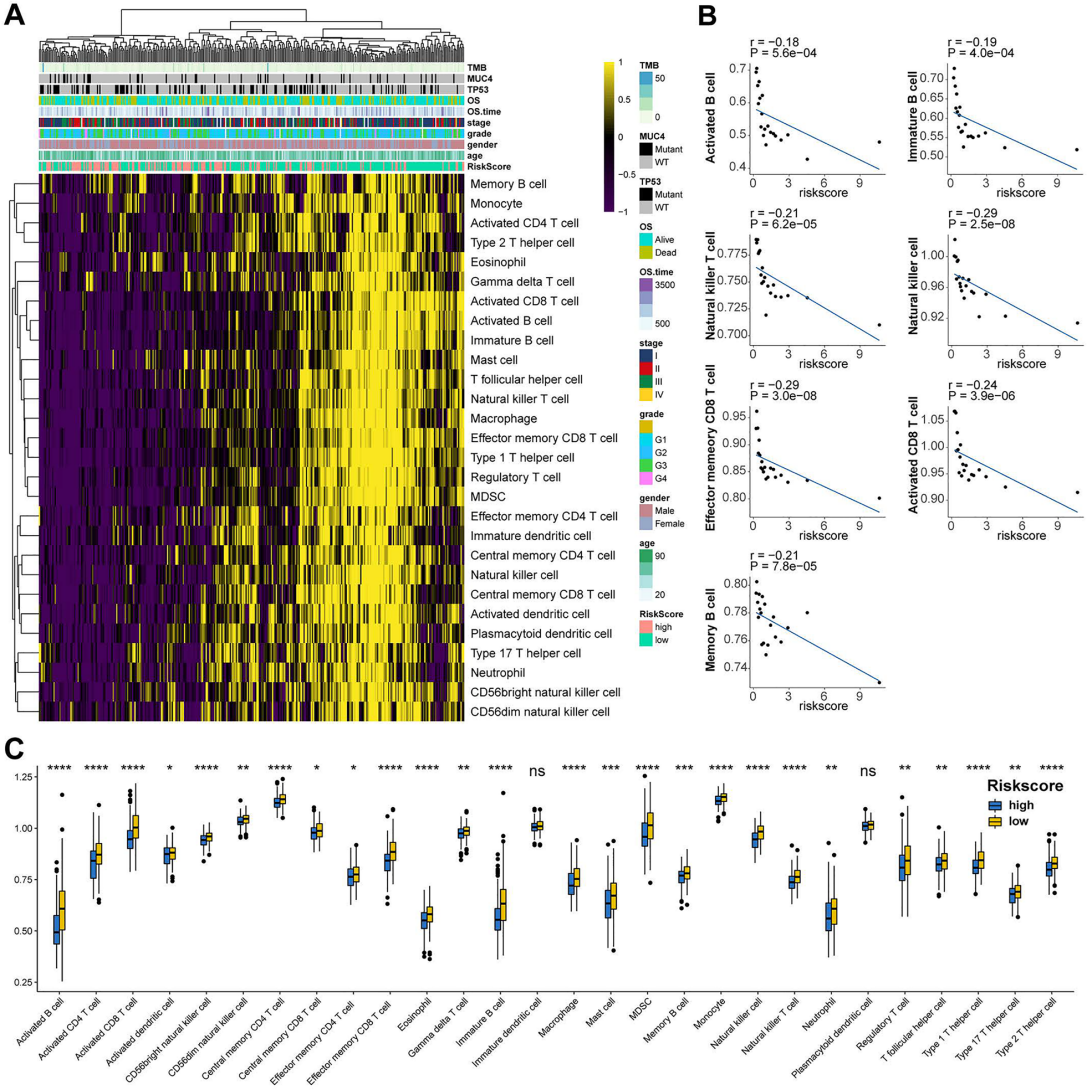
The landscape of the TME in HCC and the characteristics of high- or low IGS risk subgroups. **A**, the proportions of TME cells for 363 patients in TCGA cohort was calculated by performing CIBERSORT algorithm. Age, Gender, Grade, Stage, TP53, MUC4, TMB, OS time, and OS status were shown as patient annotations. **B**, the correlation of risk score with immune cells in TME. **C**, the proportions of TME cells in different subgroups calculated by performing ImmuCellAI. Significant statistical differences between the two subgroups were assessed using the Wilcoxon test. *ns* not significant, *P < 0.05; ** *P* < 0.01; *** *P* < 0.001; **** *P* < 0.0001

Given the robust performance of IGS risk model in prognosis for HCC, we further investigated the underlying immune-related characteristics regarding IGS genes. Based on the CIBERSORT analysis in 363 TCGA HCC patients, the differences in immune infiltration of 28 immune cell types in high- and low- IGS risk groups are illustrated in Fig. 5A. Activated B cells, Memory B cells, immature B cells, Natural killer T cells, Natural killer cells, Activated CD8 + T cells, effector CD8 T cells, Natural killer cells were abundant in the low-risk group and negatively correlated with the IGS risk score (Fig. 5B). Furthermore, we calculated the abundance of immune cells in the TME between the high- and low- IGS risk subgroups by performing ImmuCellAI analysis. As shown in Fig. 5C, patients in the high-risk group had a lower proportion of B cells, dendritic cells (DCs), central memory T (Tcm) cells, T follicular helper (Tfh) cells, CD4/CD8 T cells, CD56 natural killer (NK) cells, Eosinophil cells, neutrophils, natural regulatory T cells (nTreg), effector memory T (Tem) cells, and T regulatory type 1/2/17 (Tr1/2/17) cells, suggesting an immunosuppressive microenvironment in HCC patients with high-IGS risk.

### The prognostic value of signature in patients with anti-PD-L1 therapy

**Fig. 6:**
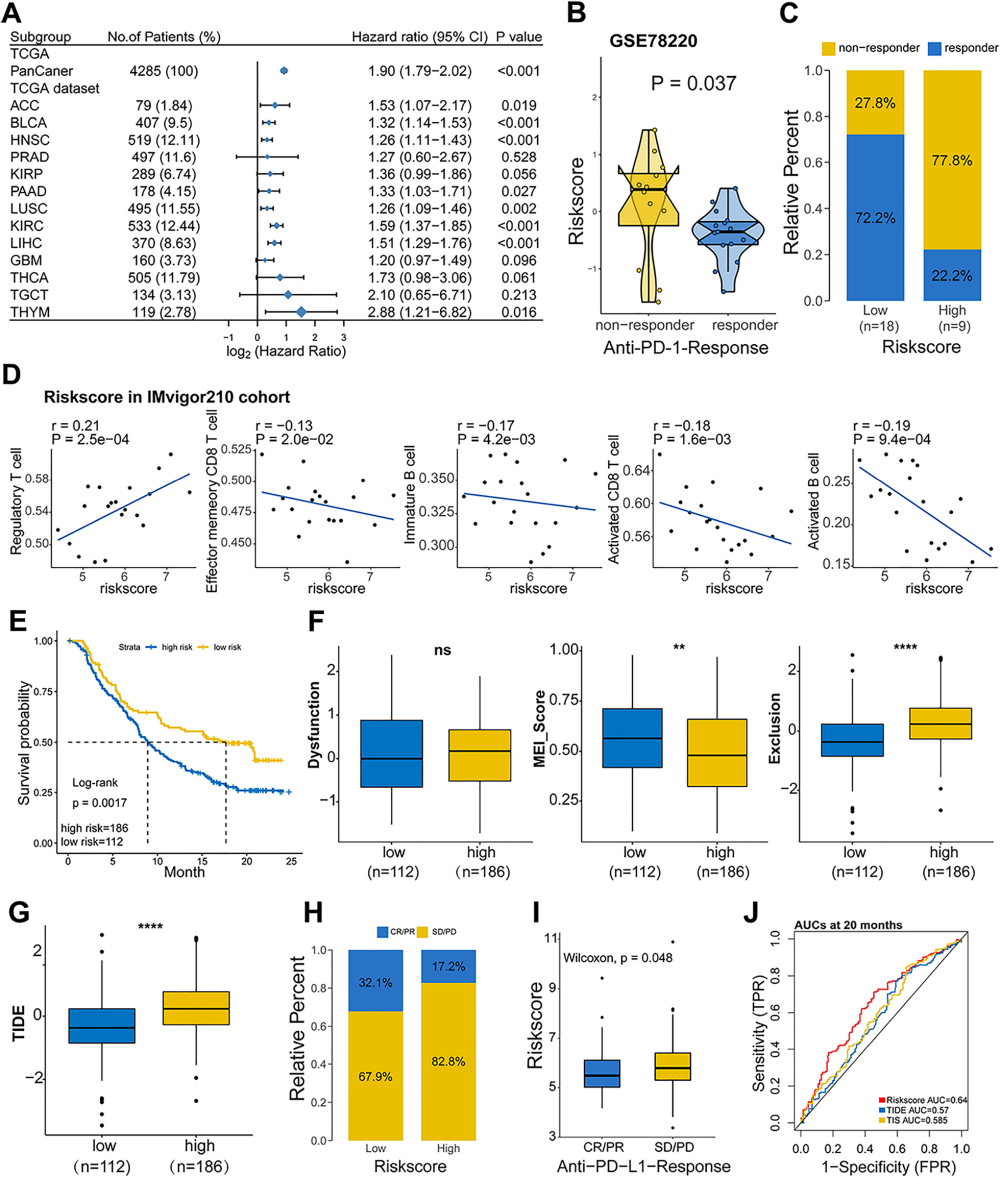
The prognostic value of signature in patients with anti-PD-L1 therapy. **A**, The prognostic value of IGS risk model in pan-cancers from TCGA datasets. **B**, Risk scores in groups with different anti–PD-1 clinical response status (non-responder: *n* = 12; responder: *n* = 15). The differences between groups were compared with the Wilcoxon test (*P* = 0.037). **C**, Rate of clinical response to anti–PD-1 immunotherapy in high- or low- IGS risk groups in the GSE78220 cohort. Responder: complete response [CR]/partial response [PR], Non-responder: stable disease [SD]/progressive disease [PD]. **D**, The correlation of IGS risk score with immune cells of TME in IMvigor210 cohort. **E**, Kaplan–Meier survival analysis of the subgroups in IMvigor210 urothelial cancer cohort. **F**, **G**, Dysfunction, MSI, T cell exclusion and TIDE score in different subgroups. **H**, Rate of clinical response to anti-PD-L1 immunotherapy in high- or low- IGS risk patients from IMvigor210 cohort (two-sided Fisher exact test, *P* < 0.05). **I**, Rate of clinical response to anti-PD-1 immunotherapy in high- or low- IGS risk groups in the IMvigor210 cohort. Responder: CR/PR, Non-responder: SD/PD. **J**, ROC curves measuring the predictive value of the IGS risk score, TIDE, and TIS at 20 months in the IMvigor210 cohort (*N* = 298). *ns* not significant, *P < 0.05; ** *P* < 0.01; *** *P* < 0.001; **** *P* < 0.0001

Then, we evaluated the prognostic value of IGS risk model in 13 independent TCGA cancer cohorts. As presented in Fig. 6A, in addition to LIHC (HR, 1.90; *P* < 0.001), IGS risk model displayed an excellent predictive capacity for cancer types such as ACC (HR, 1.53; *P* = 0.019), KIRC (HR, 1.59; *P* < 0.001), LIHC (HR, 1.90; *P* < 0.001), and THYM (HR, 2.88; *P* = 0.016). Interestingly, the IGS risk model also presented a robust prognostic value for pooled pan-cancer patients (HR, 1.90; *P* < 0.001). To further validate the potential capacity of IGS risk model, we chose a cohort administrated with anti–PD-L1 immunotherapy (GSE78220). High-IGS risk score was detected in non-responders rather than responders (Fig. 6B). In parallel, the low-IGS risk subgroup had a higher proportion of responders to anti–PD-L1 immunotherapy, suggesting a role of IGS risk score in predicting the response of PD-L1 treatment (Fig. 6C). Then we investigated the efficacy of IGS risk model in IMvigor210 urothelial cancer cohort with immunotherapy information. The IGS risk score was positively correlated with regulatory T cells, while it was negatively associated with effector memory/activated CD8 T cells and immature/activated B cells (Fig. 6D). As shown in Fig. 6E, patients with high-IGS risk score suffered from a significantly shorter OS than those with low-IGS risk score in IMvigor210 urothelial cancer cohort (*P* = 0.0017). In contrast to IGS-low subgroup, high IGS risk score led to a lower microsatellite instability (MSI) score and a higher T cell exclusion score, while there was no significant difference for T cell dysfunction (Fig. 6F). TIDE was further performed to evaluate the potential clinical efficacy of immunotherapy in different IGS subgroups. Patients with high-IGS risk score had a higher TIDE prediction score, which implied a higher possibility of immune evasion and less efficacy of ICB therapy (Fig. 6G). The ratio of clinical response to anti-PD-L1 immunotherapy was subsequently evaluated in high- or low-IGS risk subgroups in the IMvigor210 cohort. Compared with high-risk subgroup, Low-IGS risk resulted in a higher proportion of complete response [CR]/partial response [PR] and lower stable disease [SD]/progressive disease [PD] (Fig. 6H). Consistently, patients with status of SD/PD had a higher IGS risk score than patients with CR/PR (*P* = 0.048, Fig. 6I). In addition, ROC curves demonstrated that the AUC of the IGS risk score (0.64) was higher than that of the TIDE score (0.57) and TIS (0.585) at 20 months in the IMvigor210 cohort, indicating a better performance in predicting the efficacy of anti-PD-L1 immunotherapy (Fig. 6J).

### Screening potential therapeutic agents for high IGS risk HCC patients

**Fig. 7:**
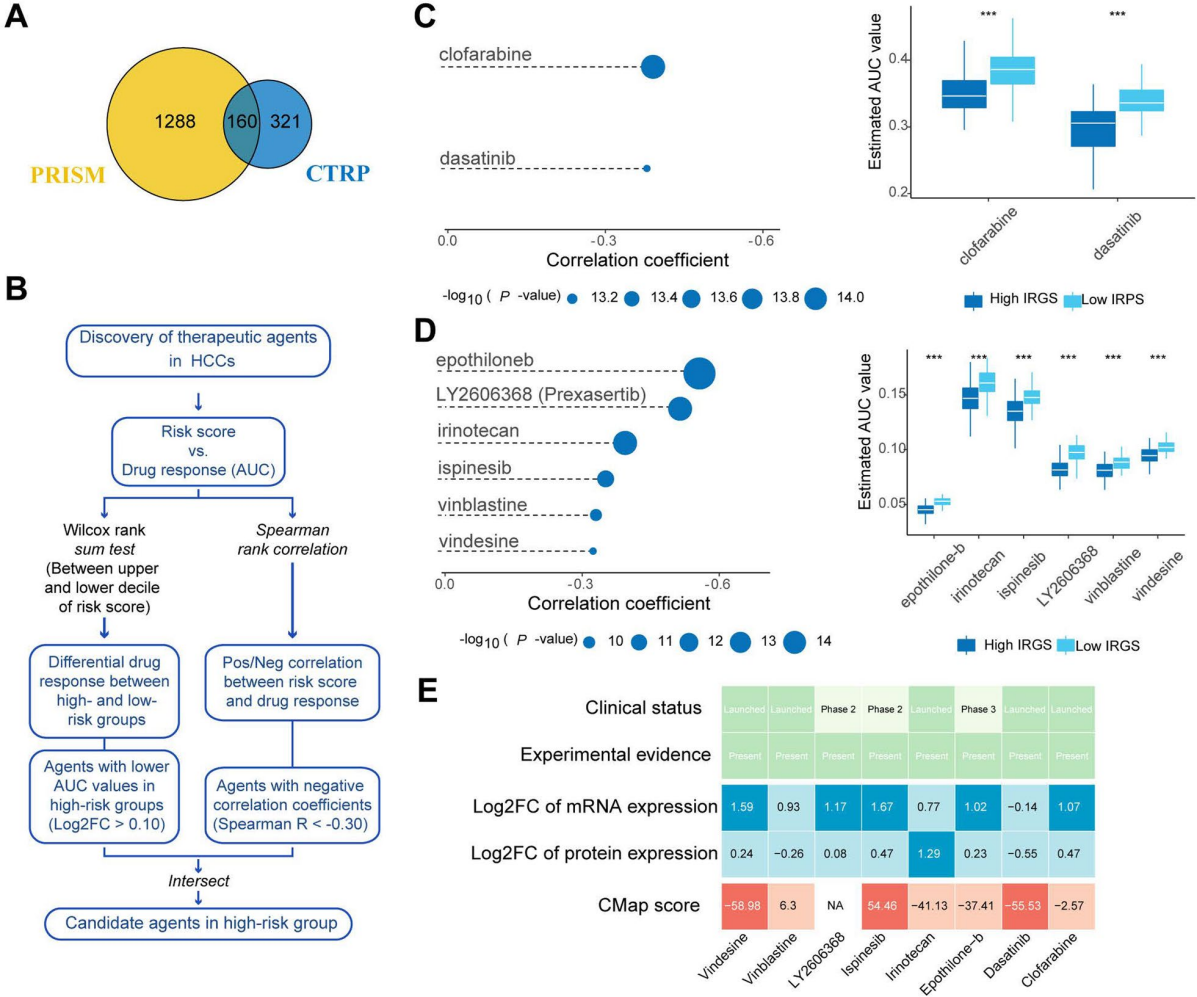
Identification of potential therapeutic agents for high IGS score sub-group. **A**, A Venn diagram for summarizing included compounds from CTRP and PRISM datasets. **B**, Schematic outlining the strategy to identify agents with higher drug sensitivity for patients with high-IGS risk score. **C**, Spearman’s correlation analysis and differential drug response analysis of 2 CTRP-derived compounds. **D**, Spearman’s correlation analysis and differential drug response analysis of 6 PRISM-derived compounds. **E**, Identification of most promising therapeutic agents for patients with high IGS-risk score according to the evidence from multiple sources

The analyses above indicated that patients with high IGS risk score might have poor prognosis and a relatively suppressive immune status. Then we tried to screen potential agents with higher drug sensitivity for high-IGS risk patients based on the CTRP and PRISM datasets. After pre-exclusion, 1929 compounds (CTRP, 481; PRISM, 1448) were included for subsequent analyses (Fig. 7A). The candidate therapeutic agents screening was conducted with a two-step strategy (Fig. 7B). First, we performed differential drug response analysis between high IGS risk score sub-group and low IGS risk score sub-group to identify candidate compounds with lower estimated AUC values in high IGS risk score patients. Next, Spearman rank correlation analysis between AUC and IGS risk score was conducted to select potential compounds with negative correlation coefficient (r <  − 0.30/CTRP or r <  − 0.30/PRISM). Based on the analyses above, we obtained the candidate agents clofarabine and dasatinib from CTRP (Fig. 7C), while 6 compounds (epothiloneb, LY2606368, irinotecan, ispinesib, vinblastine and vindesine) from PRISM (Fig. 7D). The analyses above suggested that the 8 compounds might be candidates with higher drug sensitivity in high IGS risk score patients. Furthermore, multiple perspective analyses were conducted by comprehensive evaluation of clinical status, experimental evidence, differential mRNA/protein levels of candidates’ drug targets, and CMap score. Ultimately, vindesine, ispinesib and dasatinib were identified as therapeutic agents for HCC patients with high IGS risk score (Fig. 7E).

## Discussion

**Fig. 8:**
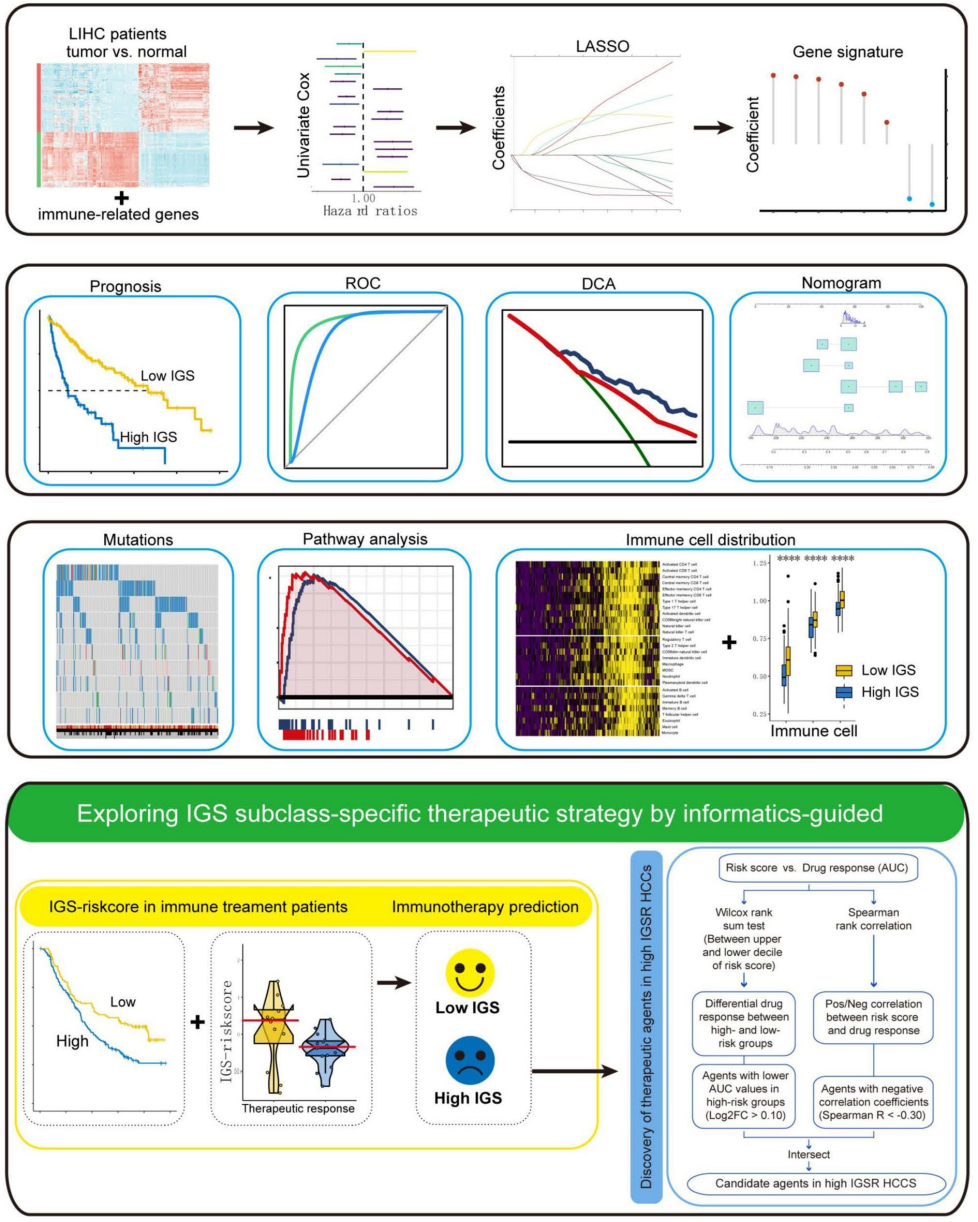
The flowchart of this study

Currently, immune-related genes-based signatures have been developed for prognosis prediction in multiple cancer types including lung cancer, gastrointestinal cancers, and breast cancer [23, 24]. These signatures were evaluated in bioinformatic datasets and multi-central cohorts, discriminating high-risk patients with poor survival, suggesting it as a promising strategy for survival prediction [25]. Nevertheless, the contribution of the immune signature to discovering oncogenic pathways, genomic alterations and immune-therapeutic response remains obscure in HCC. In the current study, we identified and validated a robust IGS through multi-step processes using multiple databases (Fig. 8). Initially, the immune-related DEGs were obtained by intersecting TCGA LIHC database and ImmPort database, and they were implicated in tumor immune and tumor microenvironment-related processes and pathways. Then, we established an immune genes-based signature to predict survival of HCC patients based on the 251 immune-related DEGs, in which 19 candidate genes were correlated with HCC prognosis. Upon these, we established an Immune-related gene signature for survival of HCC patients using LASSO Cox regression model. Subsequently, we included TCGA, ICGC, and CHCC datasets to evaluate the prognostic value of IGS for HCC patients. As expected, HCC patients with high-IGS-risk score had worse OS than patients with low-IGS-risk score in entire cohort and stratified sub-groups. In addition, IGS presented an excellent predictive performance for HCC patients in different time period follow-up survival. In consistence, the IGS score was also identified as an independent predictive factor for OS of HCC patients by Univariate and Multivariate Cox regression analyses. A nomogram including IGS risk score and clinicopathological features was generated to quantify risk assessment and survival probability for HCC patients. Compared to other factors, the nomogram exhibited profound accuracy and discrimination in survival prediction. These suggested that the IGS might be a promising factor in predicting the prognosis of HCC patients. To further validate the superiority of the IGS, we compared the predictive performance of it with previously established immune genes-based signatures in CHCC cohort. The current IGS had obviously higher AUC than any of the 4 previous signatures with improvement of NRI, indicating the better predictive accuracy for the survival of HCC patients. Besides, the pan-cancer analysis indicated that the IGS model could also predict survival for other cancer types, such as ACC, KIRC, and THYM.

It was speculated that the genomic difference might contribute to the IGS-discriminated survival of HCC patients. Thus, the genomic alterations and mutations were also analyzed and compared in different IGS groups. DEGs between high and low IGS groups were implicated in immune and metabolism-related pathways. Interestingly, the ssGSEA indicated that low-risk group was characterized by immune-activated phenotype, including IL-6/JAK/STAT3 signaling, inflammatory response and Interferon α/γ response. High-risk group was enriched in aggressive molecular changes such as DNA repair, E2F target, G2M checkpoint, and MYC targets, which might drive rapid proliferative rate and promote tumor progression. As is known, mutations of key genes facilitate the progression of HCC by inducing alterations of multiple phenotypes and pathways [26]. In addition to the genomic alterations, we also investigated the mutational status in IGS subgroups. TP53, TTN, CTNNB1 and MUC16 occupied the top mutation rates in both of IGS-high and low- groups. Of them, apart from the commonly known of TP53 and CTNNB1 in HCC, mutation of TTN and MUC16 were also observed in the progression of HCC [27, 28]. Remarkedly, ABCA13, OBSCN and SPTA1, were frequently mutated in the IGS-high group, as opposed to IGS-low group. For them, however, rare studies have elucidated the correlation of ABCA13 with HCC occurrence and development [27, 29].

Subsequently, the immune characteristics of HCC patients were evaluated with distinct IGS risk. Focusing on the immune cells in TME, high IGS risk led to a lower levels of B cells, DCs, Tcm/Tfh/Tem cells, CD4/CD8 T cells, NK cells, nTreg, and Tr1/2/17 cells, implying an immunosuppressive status in the subgroup of HCC patients. To date, limited reliable biomarkers have been developed to predict immunotherapeutic response [30]. Given that there was no immunotherapy cohort for HCC, the efficacy of IGS was further validated in different cohorts administrated with anti–PD-L1 immunotherapy to further verify its predictive capacity for immunotherapy response. Non-responders tended to have a high IGS risk, suggesting its roles in predicting the response of PD-L1 treatment. In addition, the IGS risk score was positively correlated with regulatory T cells, and negatively associated with effector memory/activated CD8 T cells and immature/activated B cells. In patients treated with ICB, IGS retained its prognostic capacity to discriminate the high-risk subset, which might benefit from immunotherapy. Moreover, high-IGS risk score led to a lower MSI score and a higher T cell exclusion score/TIDE score, suggesting a higher possibility of immune evasion and less efficacy of ICB therapy. Consistent with the immune score and prognosis, the ratio of clinical response to anti-PD-L1 immunotherapy was subsequently evaluated in high- or low- IGS risk subgroups in the IMvigor210 cohort. Higher proportion of CR/PR was observed in low-IGS risk patients with anti-PD-L1 immunotherapy. However, SD/PD status was positively associated with high IGS risk score. In addition, the IGS risk score had higher AUROC than either of TIDE score or TIS, indicating that IGS might act as a possible classifier screening the patients suited for anti-PD-L1 immunotherapy.

Nowadays, molecular targeted therapy is an attractive strategy for HCC treatment. Although numerous efforts have been made to enhance the efficacy of immunotherapy, the acknowledge of precise molecular mechanisms and available approach remain limited [31]. On the basis of aforementioned findings that high IGS risk might been correlated with poor prognosis and suppressive immune status, CTRP and PRISM datasets were used to screen potential agents with higher drug sensitivity for high-IGS risk patients. Through pre-screening and multiple perspective analyses, vindesine, ispinesib and dasatinib were identified as therapeutic agents for HCC patients with high IGS risk score. Therefore, combinational administration of vindesine, ispinesib or dasatinib may reinforce the efficacy of ICB for HCC patients with high-IGS risk. Dasatinib, a novel Src/Abl inhibitor, could suppress the growth of HCC cells and enhance the anti-HCC efficacy of irinotecan by downregulation of PLK1 synthesis [32, 33]. However, the microtubule-targeting agent vindesine has been approved in the treatment of hematological and lymphatic neoplasms. It was reported to interfere the continuous mitotic division to block the proliferation of cancer cells [34]. Though combination of vindesine and ispinesib exhibited certain anti-cancer activity [35], the effects of their alliance on HCC have not been evaluated in existed studies. It may make sense to further validate the effects of the three agents combining on enhancing immunotherapy for HCC in vitro and in vivo.

Despite of the attractive results, we have to acknowledge the fact that there were still certain limitations. As is shown above, multiple datasets and analysis approaches were included to identify and validate the efficacy of IGS. However, the sampling bias caused by heterogeneity and cross-platform integration remain in the current identification and validation. Meanwhile, further studies are needed to validate the efficacy of IGS in large size HCC cohorts and elucidate the biological functions and mechanisms underlying the gene signature in HCC.

## Conclusions

This comprehensive study provided a robust signature risk model based on the immune genes, which had profound performance in predicting the prognosis for HCC patients. In addition, patients with high IGS risk may have less response of immune therapy, while vindesine, ispinesib and dasatinib were candidate agents that improve the ICB for the high IGS risk HCC patients.

## Supplementary Information

**Additional file 1: Table S1.** Detailed information of included clinical cohorts with RNA-seq profiles. **Table S2.** The clinical characteristic information of the HCC patients in TCGA cohort. **Table S3.** The clinical characteristic information of the HCC patients in ICGC cohort. **Table S4.** The clinical characteristic information of the HCC patients in CHCC cohort. **Figure S1.** Identify the immune-related DEGs between HCC and normal liver tissues. A, Volcano plot of DEGs between HCC and normal tissues in TCGA data portal. B, Heatmap of DEGs between HCC and normal tissues. C, Venn diagram visualizing the intersections of DEGs with immune-related genes from ImmPort database. D and E, Enrichment analysis of 251 immune-related DEGs. **Figure S2.** The coefficients and the corresponding correlations. A, The lollipop map showing the coefficients of the IGS genes. B, The correlation network involving the 8 genes and risk score. **Figure S3.** Stratified survival analysis of the high- and low-risk groups in the TCGA cohort and CHCC cohort. The pooled TCGA or CHCC cohort was stratified into sub-groups based on the parameters including Age (>=60 or <60), Gender, Stage (I/II or III/IV), and TP53 status (wide-type or mutated). A, Kaplan–Meier analysis with a log-rank test was conducted in the sub-groups of the TCGA cohort. B, Kaplan–Meier curves in the sub-groups of CHCC cohort. **Figure S4.** Molecular characteristics of different IGS subgroups. A, Volcano plot of the DEGs between IGS-low and -high groups in TCGA cohort. B, GO analysis of the DEGs above. C, GSEA analysis in high IGS risk subgroup (P < 0.05, FDR < 0.25). D, GSEA analysis in low IGS risk subgroup (P < 0.05, FDR < 0.25). **Figure S5.** The correlation of IGS risk score with PDL1 and MSI genes. A, the correlation of IGS risk score with PDL1 expression in LIHC TCGA cohort. B, the expression of MSI-related genes in IGS-high and IGS-low sub-group in CHCC cohort.

## Data Availability

All datasets generated for this study are included in the manuscript.

## Abbreviations

HCC: Hepatocellular carcinoma
HBV: Hepatitis B virus
HCV: Hepatitis C virus
NASH: Non-alcoholic steatohepatitis
TACE: Transcatheter arterial chemoembolization
ICB: Immune-checkpoint blockade
CTLA-4: Cytotoxic T lymphocyte associated protein 4
ORRs: Objective response rates
TME: Tumor microenvironment
IGS: Immune-related gene signature
TCGA: The cancer genome atlas
TPM: Transcripts per million
ICGC: International Cancer Genome Consortium
CHCC: Chinese HCC
DEGs: Differentially expressed genes
ROC: Receiver operating characteristic
KM: Kaplan-Meier
ssGSEA: Single sample gene set enrichment analysis
GO: Gene Ontology
KEGG: Kyoto Encyclopedia of Genes and Genomes
CCLE: Cancer Cell Line Encyclopedia
k-NN: K nearest neighbor
NRI: Net reclassification improvement
DCs: Dendritic cells
Tcm: Central memory T
Tfh: T follicular helper
nTreg: Natural regulatory T cells
CR: Complete response
PR: Partial response
SD: Stable disease
PD: Progressive disease
