# Supplementary material for: An immune-related signature for optimizing prognosis prediction and treatment decision of hepatocellular carcinoma
Source: Eur J Med Res. 2023 Mar 15;28:123. doi: 10.1186/s40001-023-01091-w (PMC10015788; doi:10.1186/s40001-023-01091-w)
Supplement: Supplementary file 1 — Additional file 1: Table S1. Detailed information of included clinical cohorts with RNA-seq profiles. Table S2. The clinical characteristic information of the HCC patients in TCGA cohort. Table S3. The clinical characteristic information of the HCC patients in ICGC cohort. Table S4. The clinical characteristic information of the HCC patients in CHCC cohort. Figure S1. Identify the immune-related DEGs between HCC and normal liver tissues. A, Volcano plot of DEGs between HCC and normal tissues in TCGA data portal. B, Heatmap of DEGs between HCC and normal tissues. C, Venn diagram visualizing the intersections of DEGs with immune-related genes from ImmPort database. D and E, Enrichment analysis of 251 immune-related DEGs. Figure S2. The coefficients and the corresponding correlations. A, The lollipop map showing the coefficients of the IGS genes. B, The correlation network involving the 8 genes and risk score. Figure S3. Stratified survival analysis of the high- and low-risk groups in the TCGA cohort and CHCC cohort. The pooled TCGA or CHCC cohort was stratified into sub-groups based on the parameters including Age (>=60 or <60), Gender, Stage (I/II or III/IV), and TP53 status (wide-type or mutated). A, Kaplan–Meier analysis with a log-rank test was conducted in the sub-groups of the TCGA cohort. B, Kaplan–Meier curves in the sub-groups of CHCC cohort. Figure S4. Molecular characteristics of different IGS subgroups. A, Volcano plot of the DEGs between IGS-low and -high groups in TCGA cohort. B, GO analysis of the DEGs above. C, GSEA analysis in high IGS risk subgroup (P < 0.05, FDR < 0.25). D, GSEA analysis in low IGS risk subgroup (P < 0.05, FDR < 0.25). Figure S5. The correlation of IGS risk score with PDL1 and MSI genes. A, the correlation of IGS risk score with PDL1 expression in LIHC TCGA cohort. B, the expression of MSI-related genes in IGS-high and IGS-low sub-group in CHCC cohort. [file 40001_2023_1091_MOESM1_ESM.pdf]

Table S1. Detailed information of included clinical cohorts with RNA-seq profiles

Cohorts	RNA-seq/ Proteomic data	Survival data	Country	Year	Platform
TCGA-LIHC	YES/NA	Yes	USA	2017	Illumina HiSeq RNAseqV2
ICGC-LIRI-JP	YES/NA	Yes	Japan	2016	Illumina HiSeq
CHCC	YES/Yes	Yes	China	2019	Illumina HiSeq X Ten

NA, not available

Table S2. The clinical characteristic information of the HCC patients in TCGA cohort

Variable	TCGA cohort (n = 363) Number	%
**Age**		
**< = 55**	122	33.6
**> 55**	241	66.4
**Gender**		
**Female**	118	32.5
**Male**	245	67.5
**Histologic grade**		
**G1**	55	15.2
**G2**	175	48.2
**G3**	116	32
**G4**	12	3.3
**Vascular invasion**		
**None**	205	66.3
**Micro**	90	29.1
**Macro**	14	4.5
**Virus infection**		
**No**	194	53.4
**Yes**	151	41.6
**Alcohol consumption**		
**No**	229	63.1
**Yes**	116	32
**TNM stage**		
**I**	170	46.8
**II**	84	23.1
**III**	81	22.3
**IV**	4	1.1

Table S3. The clinical characteristic information of the HCC patients in ICGC cohort

	ICGC cohort (n = 229)	
**Variable**	**Number**	**%**
**Age**		
**< = 55**	25	10.9
**> 55**	204	89.1
**Gender**		
**Female**	60	26.2
**Male**	169	73.8
**Family history**		
**No**	141	65.9
**Yes**	73	34.1
**TNM stage**		
**I**	35	15.3
**II**	104	45.4
**III**	71	31.0
**IV**	19	8.3

Table S4. The clinical characteristic information of the HCC patients in CHCC cohort

	CHCC cohort (n = 159)	
**Variable**	**Number**	**%**
**Age**		
**< = 55**	88	55.3
**> 55**	71	44.7
**Gender**		
**Female**	31	19.5
**Male**	128	80.5
**Tumor number**		
**1**	117	73.6
**>1**	42	26.4
**Tumor size**		
**<=5cm**	76	47.8
**> 5cm**	83	52.2
**Liver cirrhosis**		
**No**	47	29.6
**Yes**	112	70.4
**Tumor thrombus**		
**No**	122	76.7
**Yes**	37	23.3
**AFP**		
**Low (<=300ng/ml)**	98	61.6
**High (>300ng/ml)**	61	38.4
**BCLC stage**		
**A**	68	42.8
**B**	52	32.7
**C**	39	24.5
**TNM stage**		
**I**	91	57.2
**II**	14	8.8
**III**	52	32.7
**IV**	2	1.3

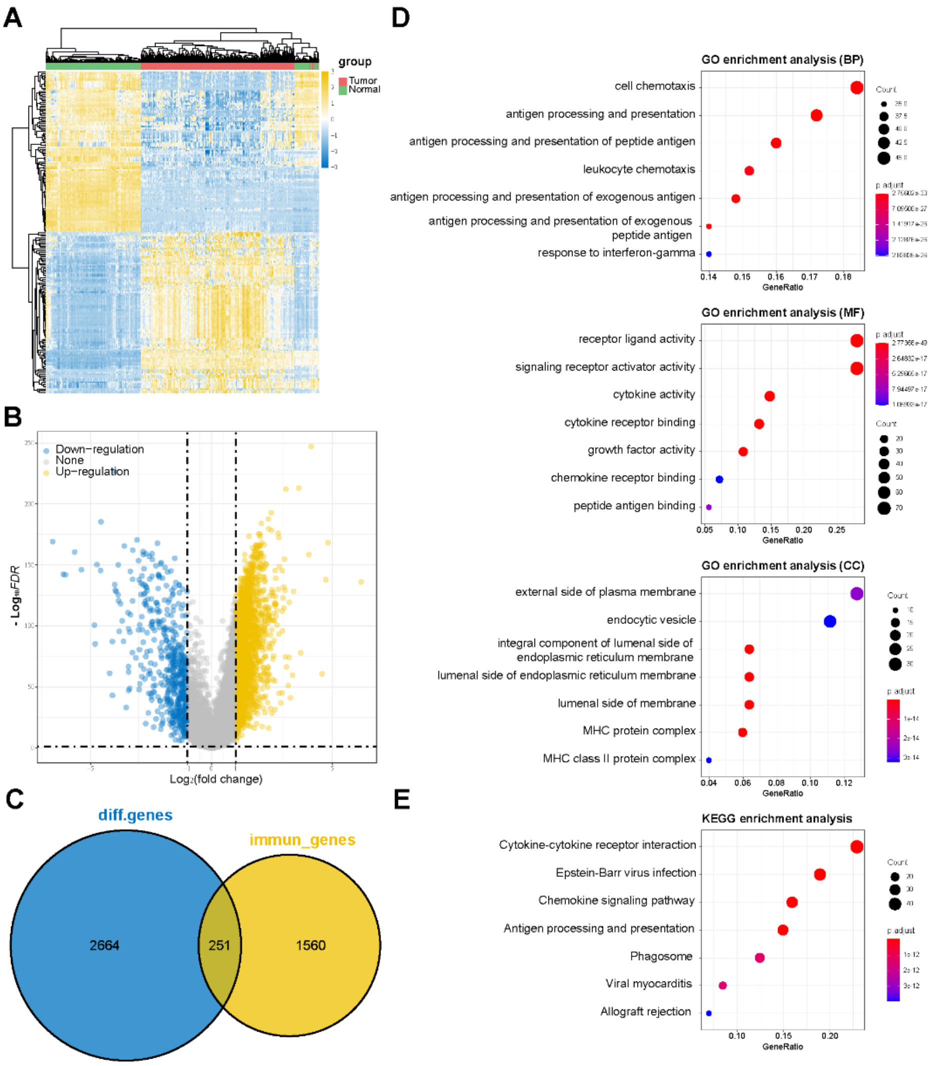

Figure S1. Identify the immune-related DEGs between HCC and normal liver tissues

A, Volcano plot of DEGs between HCC and normal tissues in TCGA data portal. **B,** Heatmap of DEGs between HCC and normal tissues. **C,** Venn diagram visualizing the intersections of DEGs with immune-related genes from ImmPort database. **D and E,** Enrichment analysis of 251 immune-related DEGs.

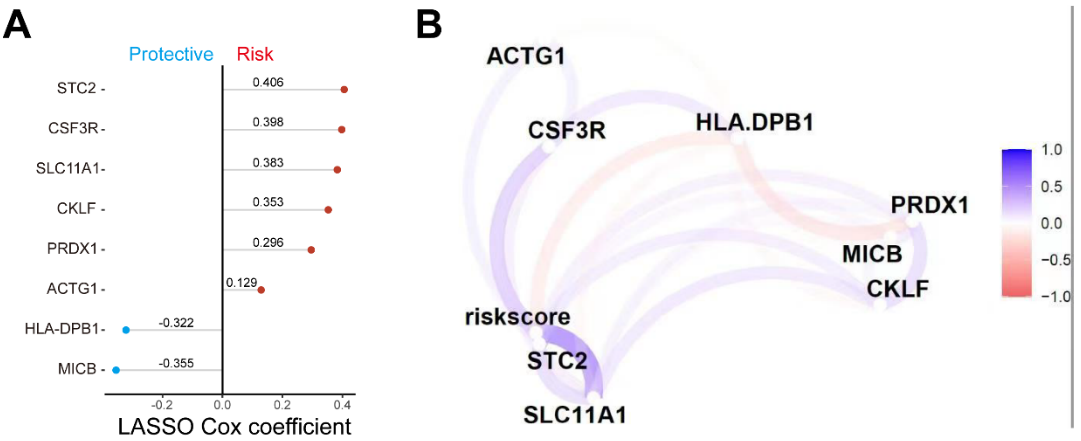

Figure S2. The coefficients and the corresponding correlations.

A, The lollipop map showing the coefficients of the IGS genes. **B,** The correlation network involving the 8 genes and risk score.

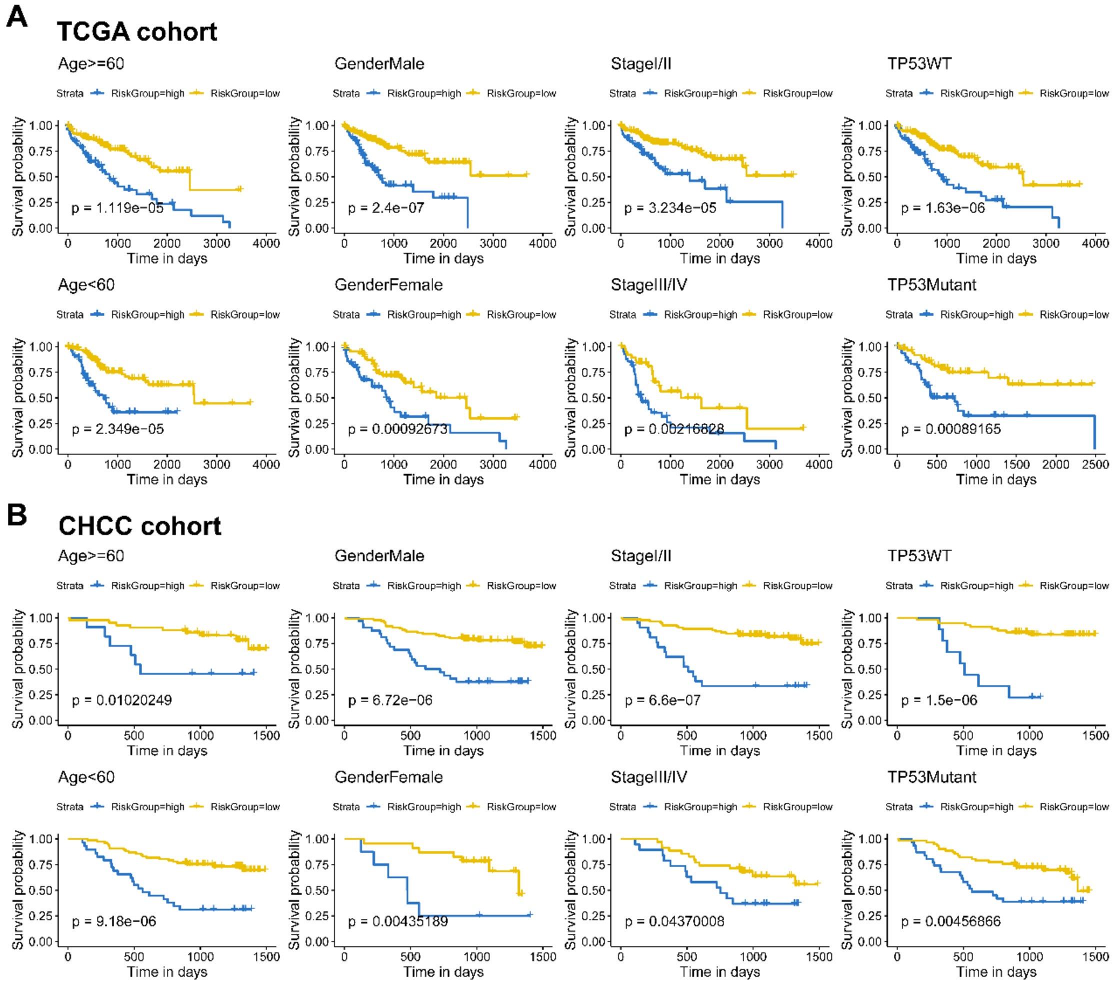

Figure S3. Stratified survival analysis of the high- and low-risk groups in the TCGA cohort and CHCC cohort.

The pooled TCGA or CHCC cohort was stratified into sub-groups based on the parameters including Age (>=60 or <60), Gender, Stage (I/II or III/IV), and TP53 status (wide-type or mutated). **A**, Kaplan-Meier analysis with a log-rank test was conducted in the sub-groups of the TCGA cohort. **B**, Kaplan-Meier curves in the sub-groups of CHCC cohort.

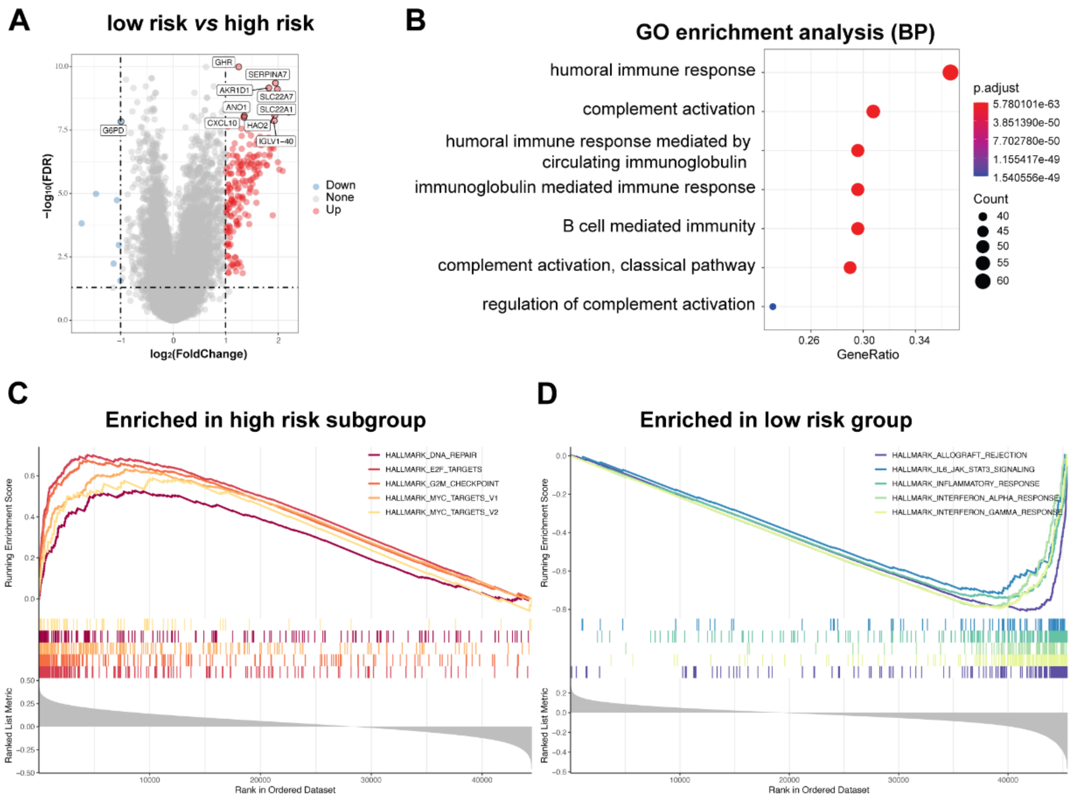

Figure S4. Molecular characteristics of different IGS subgroups. A, Volcano plot of the DEGs between IGS-low and -high groups in TCGA cohort. **B,** GO analysis of the DEGs above. **C,** GSEA analysis in high IGS risk subgroup (P < 0.05, FDR < 0.25). **D**, GSEA analysis in low IGS risk subgroup (P < 0.05, FDR < 0.25).

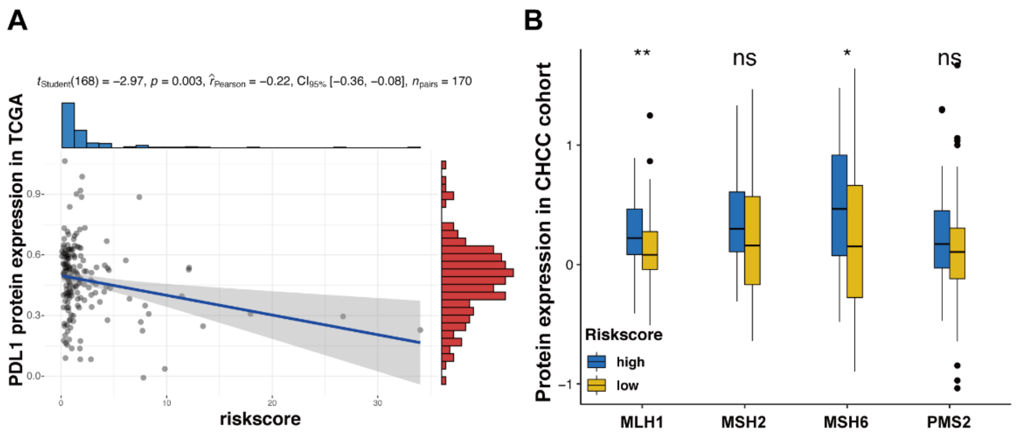

Figure S5. The correlation of IGS risk score with PDL1 and MSI genes.

A, the correlation of IGS risk score with PDL1 expression in LIHC TCGA cohort. **B,** the expression of MSI-related genes in IGS-high and IGS-low sub-group in CHCC cohort.
